# Sarcomatoid Squamous Cell Carcinoma of the Renal Pelvis Masquerading as Emphysematous Pyelonephritis with Staghorn Calculus

**DOI:** 10.1089/cren.2016.0047

**Published:** 2016-04-01

**Authors:** Dana Kivlin, Carmen Tong, Justin Friedlander, Patricia Perosio, Jay Simhan

**Affiliations:** ^1^ Department of Urology, Einstein Healthcare Network, Philadelphia, Pennsylvania.; ^2^ Department of Pathology, Einstein Medical Center Montgomery, East Norriton, Pennsylvania.

## Abstract

***Background:*** Staghorn calculi are well-established risk factors for recurrent urinary tract infections (UTIs) and subsequent renal deterioration. Less commonly, long-term urothelial irritation from a calculus may also pose a risk of malignant transformation.

***Case Presentation:*** A 77-year-old male with multiple medical comorbidities presented with a chronic right renal pelvic staghorn calculus and findings concerning for emphysematous pyelonephritis. He was subsequently taken to the operating room for a planned laparoscopic right nephrectomy. Final pathology analysis revealed sarcomatoid squamous cell carcinoma (SCC) of the renal pelvis with superimposed pyelonephritis and renal abscesses. Preoperative imaging was not suggestive of malignancy.

***Conclusion:*** Although SCC of the urothelium can be caused by chronic irritation, its presentation is usually isolated to the lower urinary tract and is rarely encountered in the renal pelvis. Our patient's presentation with sarcomatoid SCC is an even rarer entity. Chronic staghorn calculi must be considered as a potential risk factor for the development of both UTI and malignant urothelial transformation.

## Introduction and Background

Emphysematous pyelonephritis (EPN) is a severe, life-threatening necrotizing form of urinary tract infection of the kidney that is caused by gas-forming bacteria. Two major risk factors for the development of EPN include diabetes mellitus and urinary tract obstruction, which is often secondary to chronic obstructive nephrolithiasis.^1^ An additional, but highly uncommon, condition associated with chronic nephrolithiasis is the development of squamous cell carcinoma (SCC). We present the case of a 77-year-old male with end-stage renal disease (ESRD) and a chronic renal pelvic staghorn calculus who presented with findings of EPN but was ultimately found to have poorly differentiated sarcomatoid SCC of the renal pelvis.

## Presentation of Case

**FIG. 1.**
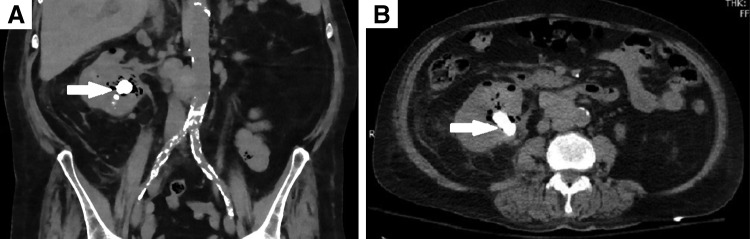
CT scan of the abdomen and pelvis without contrast in **(A)** coronal imaging and **(B)** axial imaging. The right kidney is enlarged containing a large amount of gas within the parenchyma and collecting system with a staghorn calculus in the renal pelvis measuring >3 cm (*white arrows*).

A 77-year-old Albanian male with ESRD on hemodialysis and a Karnofsky performance status of 50 presented with a history of a chronic asymptomatic right renal staghorn calculus and managed conservatively for years because of patient and family request. On initial presentation in the emergency room, the patient was seen to be lethargic, weak, and had new onset gross hematuria. His laboratory evaluations were notable for leukocytosis (17,200) and hyperkalemia (6.6 mEq/L) necessitating immediate hemodialysis. A CT scan of the abdomen and pelvis was obtained which showed a large amount of gas within the parenchyma and collecting system of an enlarged right kidney, consistent with EPN (Fig. 1). After medical optimization, the patient was taken to the operating room for a planned laparoscopic right nephrectomy. The kidney was found to be encircled by an inflammatory rind and there was bulky lymphadenopathy partially encasing the inferior vena cava. Upon manipulation of the kidney during nephrectomy, the patient developed septic shock necessitating maximal dosing of three vasopressors. Hilar control was, therefore, established expeditiously through en bloc stapling using a 60-mm Endo-GIA device (Covidien, Minneapolis, MN).

**FIG. 2.**
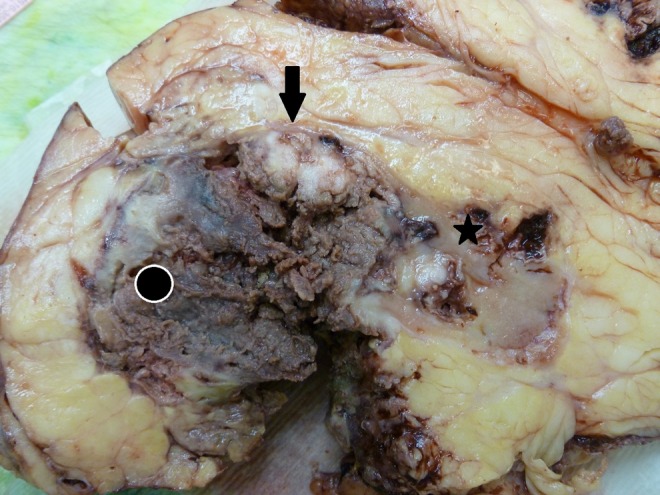
Gross appearance of kidney that has been largely destroyed by a necrotic abscess and tumor centrally (*black circle* with *white edges*). More viable tumor is seen peripherally as solid tan nodules (*black arrow*). A small amount of normal renal cortex and medulla remain (*black star*).

**FIG. 3.**
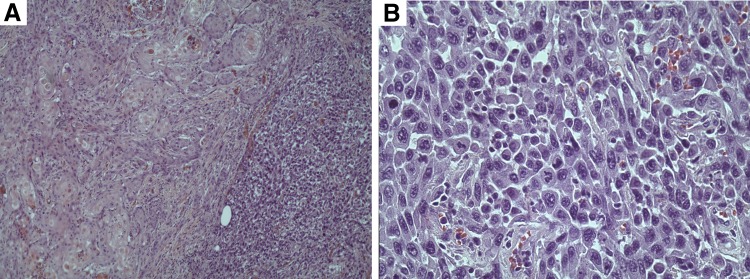
**(A)** Microscopic appearance of squamous cell carcinoma transitioning to an area of poorly differentiated sarcomatoid carcinoma. **(B)** High power magnification demonstrating hyperchromatic and pleomorphic spindle cells representing sarcomatoid carcinoma.

Upon gross examination of the specimen, normal parenchyma had been replaced by a large, friable necrotic mass, measuring 10 cm, and the pelvocaliceal system could not be identified (Fig. 2). Microscopically, there was extensive parenchymal necrosis with areas of large geographic abscesses. Further examination of the mass revealed poorly differentiated sarcomatoid SCC of the renal pelvis with a background of chronic pyelonephritis, extensive necrosis, and abscess formation. Histologic sections of the pelvis showed remnants of pelvocaliceal lining with squamous carcinoma *in situ* transitioning to invasive SCC (Fig. 3A). Multiple areas of the tumor also demonstrated poorly differentiated sarcomatoid features, composed of hyperchromatic and pleomorphic spindle cells (Fig. 3B).

The patient was admitted to the ICU after nephrectomy, where his condition stabilized with supportive care. As septic shock resolved, he slowly recovered to his baseline functional status. His preoperative urine cultures grew >100 K of *Klebisella pneumoniae*. The tissue cultures grew pan sensitive *Escherichia coli* in addition to *K. pneumoniae*. Blood cultures remained negative. Due to the diagnosis of this advanced malignancy, the patient was evaluated by medical oncology during his hospital stay and a family discussion was convened regarding goals of treatment. Ultimately, a conservative approach was initiated, with eventual discharge to a rehabilitation center after a total length of stay of 39 days.

## Discussion

Primary upper urinary tract tumors are extremely rare, with an incidence of 5% to 10% of all urothelial malignancies.^2^ Although the majority of these tumors are urothelial in nature, SCC accounts for <10% of upper tract malignancies and these cancers usually present at an advanced stage.^2^ Commonly associated with chronic irritation and inflammation, patients with SCC usually present with long-standing obstruction from calculi that are present in as much as 50% of cases.^1^ Malignancy is often challenging to diagnose before treatment as routine radiologic investigations rarely demonstrate any signs of cancer. In one characteristic report, Jain and colleagues presented a series of four patients with SCC of the renal pelvis associated with staghorn calculi, only one of which had suggestion of malignancy on preoperative imaging.^3^ Although survival is variable and is often dependent on cancer stage, tumor grade, and patient-related factors, SCC generally carries a poor prognosis.^4,5^ In the largest series of reported cases of SCC of the renal pelvis, median survival was 11.3 months and only 5 out of 12 patients were alive at 30 months.^5^ Due to the rarity of this malignancy, response to surgery, chemotherapy, or radiation therapy has not been studied extensively but has anecdotally provided only minimal survival benefit.

Sarcomatoid SCC of the renal pelvis is an even more uncommon entity with only a few reported cases in the literature.^6,7^ Although rare in the renal pelvis, this uncommon tumor with carcinomatous and sarcomatous features can occur in many locations, most commonly the upper aerodigestive tract.^8^ Risk factors in the urinary tract are similar to that of pure SCC, including chronic irritation, inflammation, and nephrolithiasis. With a rapidly progressive tumor biology, these cancers also pose a high tendency to recur and metastasize to distant regions such as the liver and lungs^9^ In one of the only other reports of this pathologic variant, Kandemir and associates described an unfortunate case of sarcomatoid SCC of the renal pelvis with liver metastasis in a 26-year-old patient with bilateral urolithiasis and hydronephrosis who died a mere days after nephrectomy.^6^

Understanding the biologic, diagnostic, and therapeutic implications of sarcomatoid SCC in other organ systems may provide valuable insight toward patients presenting with renal pelvic pathology. As such, these highly rare malignancies of the renal pelvis have been more extensively studied in the fields of otolaryngology and gynecology. The most widely accepted pathogenesis is through both squamous cell and spindle cell tumors sharing similar molecular and immunohistochemical features.^9^ The histopathologic diagnosis generally depends on demonstration of the tumor showing areas of pure SCC with transition zones where spindle cells predominate, as in our patient.^9^ In tumors where a spindle cell component is more prominent, the pathologic diagnosis may be challenging and immunohistochemistry studies may be of added utility. Tumors that stain positive for at least one cytokeratin will favor the diagnosis of sarcomatoid SCC over pure sarcomas.^10^

## Conclusion

Malignant transformation of renal pelvis urothelium is a rare occurrence, but can be associated with long-standing nephrolithiasis as well as from chronic irritation and inflammation. Urologists managing patients with an observational strategy for staghorn calculi should be aware of this small but potentially devastating risk.

## Abbreviations Used

CT: computed tomography
EPN: emphysematous pyelonephritis
ESRD: end stage renal disease
SCC: squamous cell carcinoma
UTI: urinary tract infection

## References

[1] Somani BK, Nabi G, Thorpe P, et al. Is percutaneous drainage the new gold standard for the management of emphysematous pyelonephritis? Evidence from a systematic review. J Urol 2008;179:1844–1849 18353396 10.1016/j.juro.2008.01.019

[2] Rouprêt M, Babjuk M, Compérat E, et al. European guidelines on upper tract urothelial carcinomas: 2013 Update. Eur Urol 2013;63:1059–1071 23540953 10.1016/j.eururo.2013.03.032

[3] Jain A, Mittal D, Jindal A, et al. Incidentally detected squamous cell carcinoma in renal pelvis in patients with staghorn calculi: Case series with review of the literature. ISRN Oncol 2011;2011:1–6 10.5402/2011/620574 PMC3200069 22091426

[4] Holmäng S, Lele SM, Johansson SL Squamous cell carcinoma of the renal pelvis and ureter: Incidence, symptoms, treatment and outcome. J Urol 2007;178:51–56 17574059 10.1016/j.juro.2007.03.033

[5] Busby JE, Brown GA, Tamboli P, et al. Upper urinary tract tumors with nontransitional histology: A single-center experience. Urology 2006;67:518–523 16527570 10.1016/j.urology.2005.09.010

[6] Kandemir O, Tatslişen A, Kontaş O, et al. Sarcomatoid squamous cell carcinoma of the right renal pelvis with liver metastasis: Case report. J Urol 1995;153:1895–1896 7752344

[7] Shimasaki N, Inoue K, Nishigawa H, et al. Combined small cell carcinoma and sarcomatoid squamous cell carcinoma in the renal pelvis. Int J Urol 2005;23:686–689 16045564 10.1111/j.1442-2042.2005.01134.x

[8] Oktay M, Kokenek-Unal TD, Ocal B, et al. Spindle cell carcinoma of the tongue: A rare tumor in an unusual location. Patholog Res Int 2011;2011:572381 21403898 10.4061/2011/572381 PMC3043295

[9] Anderson CC, Le BH, Robin-Bennett B Sarcomatoid squamous cell carcinoma. In: Li X (ed). Squamous Cell Carcinoma. Croatia: InTech, 2012, pp. 53–66

[10] Dimitriou RJ, Gattuso P, Coogan CL Carcinosarcoma of the renal pelvis. Urology 2000;56:508 10962328 10.1016/s0090-4295(00)00660-9

